# Abismal: Approximate Bayesian Inference for Scaling and Merging at Advanced Lightsources

**DOI:** 10.1063/4.0000945

**Published:** 2025-10-27

**Authors:** Doris Mai, Doeke R Hekstra, Frédéric Poitevin, Kevin M Dalton

**Affiliations:** 1 Machine Learning and Computer Vision Group, LCLS Data Systems, SLAC National Accelerator Laboratory, Menlo Park, CA, USA; 2 Department of Molecular & Cellular Biology, Harvard University, Cambridge, MA, USA; 3 Department of Biology, New York University, New York, NY, USA

## Abstract

Experimental artifacts in diffraction data are typically corrected by an optimization algorithm known as scaling. After appropriate scaling, redundant measurements can then be merged. Recently, it was shown that unifying the two procedures, scaling and merging, into a single algorithm based on Bayesian (variational) inference and deep learning compared favorably to other programs for a broad range of experimental designs (Dalton et al., 2022). However, the memory requirements of this method pose challenges in serial crystallography and other data- intensive applications. In this talk, I will highlight the algorithmic innovation in the new software Abismal that addresses this limitation while preserving the benefits of the unifying Bayesian approach. Additionally, I will discuss ongoing efforts toward deploying and maintaining the software at LCLS and other light sources, including supporting a user-friendly interface with cctbx.xfel for automated workflows during beam time. By leveraging advances in machine learning, Abismal facilitates recovering structural signals in a robust and efficient way from high-throughput experiments with hundreds of thousands to millions of diffraction images.

